# Psychometric properties of the 7-item game addiction scale among french and German speaking adults

**DOI:** 10.1186/s12888-016-0836-3

**Published:** 2016-05-10

**Authors:** Yasser Khazaal, Anne Chatton, Stephane Rothen, Sophia Achab, Gabriel Thorens, Daniele Zullino, Gerhard Gmel

**Affiliations:** Department of Mental health and psychiatry, Geneva University Hospitals, Geneva, Switzerland; Department of psychiatry, Geneva University, Geneva, Switzerland; Lausanne University, Lausanne, Switzerland

**Keywords:** Internet addiction, Internet Gaming Disorder, Game Addiction Scale

## Abstract

**Background:**

The 7-item Game Addiction Scale (GAS) is a used to screen for addictive game use. Both cross cross-linguistic validation and validation in French and German is needed in adult samples. The objective of the study is to assess the factorial structure of the French and German versions of the GAS among adults.

**Methods:**

Two samples of men from French (*N =* 3318) and German (*N =* 2665) language areas of Switzerland were assessed with the GAS, the Major Depression Inventory (MDI), the Brief Sensation Seeking Scale, and the Zuckerman-Kuhlman Personality Questionnaire (ZKPQ-50-cc). They were also assessed for cannabis and alcohol use.

**Results:**

The internal consistency of the scale was satisfactory (Cronbach α = 0.85). A one-factor solution was found in both samples. Small and positive associations were found between GAS scores and the MDI, as well as the Neuroticism-Anxiety and Aggression-Hostility subscales of the ZKPQ-50-cc. A small negative association was found with the ZKPQ-50-cc Sociability subscale.

**Conclusion:**

The GAS, in its French and German versions, is appropriate for the assessment of game addiction among adults.

**Electronic supplementary material:**

The online version of this article (doi:10.1186/s12888-016-0836-3) contains supplementary material, which is available to authorized users.

## Background

The expansion of the Internet comes with numerous benefits, including its use for commercial, social, psychological, academic, and medical purposes [1–9]. Serious concerns have been raised, however, related to possible Internet and Internet Gaming addictions [10–15]. In particular, online games have received attention for their possible links to addictive patterns of use in a subset of users [16–18]. A number of studies have reported important associations between Internet or game addictions and psychiatric constructs or disorders [19], such as depression [20–22], anxiety disorders [22, 23], attention deficit disorder [21, 24], loneliness [25–27], introversion, neuroticism, impulsivity [17, 18, 26, 28–31], and substance abuse disorders [32]. Excessive Internet use has furthermore been associated with family and social problems [33, 34].

Internet gaming disorder” (IGD) [35] was introduced in the section 3 of the DSM-5 as a condition warranting more clinical research and experience before it might be considered for inclusion as a formal disorder. The DSM-5 suggests that IGD may refer to the persistent and recurrent use of Internet games associated with distress or impairment in a minimum 12-month period.

It was commonly reported that symptoms of Internet Gaming Disorder include persistent preoccupation with Internet Gaming, difficulty to control or cut down the time spent on games, negative consequences of loss of control (deceiving others, conflict, social isolation and fatigue, lost relationship or opportunities), loss of interest in other activities, use of the Internet gaming to escape or relieve a dysphoric mood, withdrawal and tolerance [36–38].

Since the emergence of the concept of Internet addiction [39] and Internet Gaming Disorder, a number of psychometric measures have been developed [37, 39–43]. The 7-item Game Addiction Scale (GAS) is one such short measure. This scale was specifically developed by Lemmens et al. to assess gaming among adolescents [44] and was conceptually based on the criteria for pathological gambling in the fourth edition of the DSM (DSM-IV). Each item on the GAS is preceded by the statement “During the last six months, how often…” and is scored on a 5-point Likert scale (1 = never, 2 = rarely, 3 = sometimes, 4 = often, and 5 = very often). Lemmens et al. [44] suggested two formats for the assessment of the presence of game addiction: a monothetic format (all items scoring above 3) and a polythetic format (at least half of the items scoring 3 or above). He hypothesized that the monothetic format would lead to a better estimate of the prevalence of addiction than the polythetic format would [44].

Good correlations were found between GAS scores and the weekly time spent on games. The scores were furthermore correlated with a number of constructs previously associated with game addictions such as lower life satisfaction, lower social competence, higher loneliness, and higher aggression [44]. Higher GAS scores were associated with attentional bias and more errors in response inhibition related to game cues [45]. The findings are in line with numerous studies linking impulsivity and specific cue reactivity with other addictive behaviors [46–48], Internet addiction [17, 29] or gambling-related disorders [49]. Factorial analyses indicated that the GAS was unidimensional [44, 50]. As compared with other scales, the GAS has better coverage of the IGD criteria in the DSM-5 [35] (see also Table 1).

**Table 1:** GAS and its concordance with the DSM-5 proposed criteria for Internet gaming disorder

	How often in the last six months…	Internet gaming disorder: proposed criteria (DSM-5)^a^
Item 1	Have you thought all day long about playing a game ?	Preoccupation (item 1)
Item 2	Have you played longer than intended?	Withdrawal (item 5)
Item 3	Have you played games to forget about real life?	Tolerance (item 2)
Item 4	Have others unsuccessfully tried to reduce your time spent on games ?	Unsuccessful attempts to reduce or stop (item 4)
Item 5	Have you felt upset when you were unable to play ?	Loss of interests in other activities (7)
Item 6	Have you had arguments with others (e.g. family, friends) over your time spent on games ?	Continue despite problems (item 7)
Item 7	Have you neglected important activities (e.g. school, work, sports) to play games?	Deceived others (item 6)
		Escape (item 3)
		Lost relationships or opportunities

^a^The suggested GAS items for each criteria are shown in parentheses. Number of criteria: 5 or more. Time criteria: 12 months

Surprisingly, the psychometric characteristics of the scale were not reported among young adults despite the wide dissemination of games in that population [16], particularly among young males [25].

The main goal of the present study was to investigate the psychometric properties of the 7-item GAS in young adult males. A secondary goal of the study was to perform a cross-validation of two samples from different linguistic regions in Switzerland—French- and German-speaking—and to assess the invariance or the equivalence property of the GAS across these two linguistic groups.

## Methods

### Participants and procedure

The data used in this study originated from a longitudinal study designed to assess substance and game use among young Swiss men: the Cohort Study on Substance Use Risk Factors (C-SURF).

The study at hand, issued from the C-SURF research protocol number 15/07, was approved by the Lausanne University Medical School’s Ethics Committee for Clinical Research.

All participants gave their written informed consent to participate in the study.

Participants were recruited between August 2010 and November 2011 in three of the six national army recruitment centers. One of the centers is located in Lausanne (French-speaking area) and the other two in Windisch and Mels (German-speaking area). The recruitment centers cover all Swiss French-speaking cantons and 21 of 26 cantons in Switzerland. Army conscription is mandatory in Switzerland, and so virtually all young men of the corresponding cantons who are about 20 years old were eligible for participation in the C-SURF study.

During the study recruitment period, 15,074 men reported to the recruitment centers. Of these potential participants, 1,829 (12.1 %) were never informed about C-SURF (brief illness at the appointment time, not informed about the study by the military staff), or were randomly selected into another ongoing study, called CH-X [51]. CH-X is a repeated cross-sectional survey, which has a fixed and mandatory schedule of 90 min within the recruitment procedures. Hence, commonly participation in CH-X did not interfere with our enrolment procedures, which took place before the start of army procedures. However, in few cases participants were already gone to fill in CH-X questionnaires before we could inform them about our study. As we have promised not to interfere with army procedures, we were not able to contact some of them. To the best of our knowledge, we cannot see any systematic biases that these few non-contacted people due to CH-X requirements may have caused. These men did not report to the research staff and could not be included. Of the 13,245 (87.9 %) men who were informed about the study, 7,563 (57.1 %) gave their written consent to participate. Unfortunately, we have no information on the motives for not consenting. One reason may be that signing a kind of contract for a long-lasting study (C-SURF is planned for a period of 10 years) may deter some individuals. A comparison of consenters and non-consenters [52] revealed that non-consenters were more often substance users than consenters, but differences were often non-significant and sometimes in opposite direction (e.g. consenters were more often alcohol users than non-consenters). Recruitment centers were used only to enroll participants; questionnaires were sent to private addresses and confidentiality was assured, particularly regarding the army. A final total of 5,990 (79.2 %) participants completed the baseline questionnaire. Of this number, 3,320 were French speaking and 2,670 were German speaking.

### Instruments

#### Game addiction scale (GAS)

The English version of the scale was translated and back-translated to French and German. An introductory statement for the scale items clearly directed participants to answer in relation to their game use: “Now we are interested to know how much time you have spent on games. This includes cybergames on internet or games on a console” (Additional file 1).

In accordance with the hypothesis of Lemmens et al. [44], those who scored “sometimes” or more on all seven items were defined as monothetic gamers (“pathological gaming”), and those who scored “sometimes” or more on at least half of the items (four to six of seven items) were defined as polythetic gamers (excessive gaming).

High reliabilities for the Game addiction scale with Cronbach alpha of .82 to .87 were reported in the original validation study [44].

#### Major depression inventory (MDI)

The MDI was used to determine the level of depression in the past two weeks [53, 54]. It is a self-report mood questionnaire. A six-point scale from “never” (0) to “all the time” (5) was used, and a total score was computed. The MDI can also be used as a diagnostic instrument with algorithms leading to the DSM-IV or to the International Classification of Mental and Behavioral Disorders (ICD-10) categories of no depression, mild to moderate depression, and severe depression.

Previous studies on the Major Depression Inventory indicate that MDI has good reliability and internal consistency (Cronbach’s alpha coefficient: up to 0.94) as well as good sensitivity, specificity, and validity as a unidimentional depression severity scale with adequate cut-off scores [53, 55, 56].

#### Brief sensation seeking scale (BSSS)

The BSSS [57] is an eight-item scale, each item scored on a five-point scale from “strongly disagree” (1) to “strongly agree” (5). The BSSS involves the following dimensions: adventure, boredom, disinhibition, and experience seeking. The total score was previously associated with a risk of drug use in a sample of adolescents [57].

Adequate internal consistency of the BSSS was previously reported (Cronbach’s alpha coefficient: 0.74) [57].

#### The Zuckerman-Kuhlman personality questionnaire (ZKPQ-50-cc)

The ZKPQ-50-cc assesses different aspects of personality [58]. Three subscales, each consisting of 10 items, were used to assess neuroticism/anxiety, sociability, and aggression/hostility. The participants indicated whether they agreed or disagreed with each statement. A mean score was computed for each subscale. Other studies have shown a contribution of neuroticism/anxiety and aggression/hostility to Internet addiction [59]. The ZKPQ-50-cc showed satisfactory psychometric and cross-cultural properties, including adequate reliability across subscales and countries (Cronbach’s alpha coefficient up to 0.70) [58].

#### Questionnaires on substance use

Alcohol use was assessed in a 12-month time frame (Table 2). Accordingly, the frequency of binge drinking (six standard drinks or more on one occasion) and of drinking days during the week (Monday to Thursday) was calculated. Age of onset of drunkenness (first episode of being drunk) was also assessed according to the European School Survey Project on Alcohol and Other Drugs [60]. Cannabis use was assessed by asking about the following: age of cannabis use onset, age of first “high” on cannabis, and cannabis use and frequency of use during the past 12 months.

**Table 2:** Participants’ characteristics

	French-speaking community (*N =* 3,318)	German-speaking community (*N =* 2,665)
Age	20.3 (1.3)	19.7 (1.1)
Age first drunk	15.6 (1.8)	15.4 (1.7)
Frequency six standard drinks or more (binge) on one occasion in the past 12 months		
- Never	23.8	17.8
- Less than monthly	31.4	34.7
- Once a month or more	44.8	47.5
Frequency of drinking during the week (Monday to Thursday) in days per week in the past 12 months	27.3 (42.4)	26.2 (41.7)
Age cannabis use onset	15.9 (2.0)	15.6 (1.9)
Age you got “high” on cannabis for the first time	15.9 (2.0)	15.7 (1.8)
Cannabis use last 12 months		
- Yes	32.0	28.9
- No	68.0	71.1
Cannabis frequency of use last 12 months		
- Once a month or less	16.7	16.3
- Once to 3 times per week	7.7	7.3
- 4 to 5 times per week or more often	7.6	5.3
- Never	68.0	71.1
Gaming use classification		
- Monothetic	2.3	2.3
- Polythetic	10.6	8.1
- Non-problematic game users	87.1	89.6
Major Depression Inventory		
- No depression	93.3	94.5
- Mild to moderate depression	4.1	3.5
- Severe depression	2.6	1.8
Brief Sensation Seeking (BSSS total scale)		
- Adventure subscale	2.9 (0.9)	3.2 (0.9)
- Boredom subscale	2.8 (1.2)	3.1 (1.2)
- Disinhibition subscale	2.7 (1.0)	3.1 (1.0)
- Experience seeking subscale	2.9 (1.1)	3.0 (1.1)
Personality ZKPQ-50-cc	3.2 (1.2)	3.6 (1.1)
- Aggression/hostility subscale	4.3 (2.3)	4.0 (2.1)
- Anxiety subscale	2.1 (2.1)	1.9 (1.9)
- Sociability subscale	5.9 (2.3)	5.9 (2.1)

Data are expressed as mean and standard deviation (M [SD]) or as a percentage depending on their quantitative or categorical nature

### Statistical analyses

In this study, we used SPSS 18.0 and AMOS 19.0 (Analysis of Moment Structures; SPSS Inc., Chicago, IL) software programs. First, descriptive statistics were computed for the participants’ characteristics. Internal consistency, that is, the extent to which the GAS items were interrelated, was then measured by using Cronbach’s coefficient. Streiner and Norman [61] suggest that alpha be above 0.70, but not much higher than 0.90.

Next, exploratory factor analyses (EFAs) were used to assess factor stability of the scale as validated by Lemmens and al [44]. The number of factors were extracted with Velicer’s minimum average partial (MAP) test performed on the correlation matrix [62]. This number was then confirmed through parallel analyses. In parallel analyses, the focus is on the number of components that account for more variance than the components derived from random data, whereas in the MAP test, the focus is on the relative amounts of systematic and unsystematic variance remaining in a correlation matrix after extractions of an increasing number of components [63].

Although EFA is more appropriate for newly designed questionnaires, it is not uncommon to also use it in a revalidation process when data are collected from another sample or another population. The use of EFA here was to evaluate the stability of the factors in the two linguistic regions, as this is a basic prerequisite for further investigation of the equivalence of the tool among the different subgroups.

For the determination of multigroup invariance, we used the procedure described in the structural equation modeling (SEM) following the work of Jöreskog [64]. In testing for group equivalence, it is customary to use confirmatory factor analysis (CFA) models, a method among the general class of SEM. Depending on the research question, searching for group equivalence may imply a series of tests performed in the following restrictive order: configural equivalence, measurement equivalence, and structural equivalence. Configural invariance testing focuses on the extent to which the number of factors and patterns of their structure are similar between groups. Worth noting, however, is that determination of an appropriate baseline model is required for each group separately, upon which the configural model is derived. On the other hand, in testing for measurement and structural invariance, interest focuses more specifically on the extent to which parameters in the measurement and structural components of the model are equivalent across the groups [65, 66]. Given that our research questions concern measurement equivalence across groups, the statistical analyses focus on configural invariance and invariance of factor loadings across the two linguistic regions.

#### Evaluation of model fit

Goodness of fit of the models is examined through various indices, as described below [67].

- The *χ*^2^ to degrees of freedom ratio (*χ*^2^/df). Several researchers have recommended the use of this ratio as a measure of fit to overcome problems associated with the *χ*^2^ test statistic. These problems include, among others, violation of assumptions, model complexity, and dependence on sample size. Ratios as low as 2 seem to indicate a reasonable fit.
- The comparative fit index (CFI). The CFI ranges from 0 to 1, with higher values indicating better fit. A rule of thumb is that values greater than 0.95 may be interpreted as a good fit, whereas values between 0.90 and 0.95 are indicative of acceptable fit relative to the independence model.
- The root mean square error of approximation (RMSEA). This is a measure of approximate fit in the population and is therefore concerned with the discrepancy due to approximation. The RMSEA is bounded below 0. RMSEA values less than or equal to 0.05 can be considered as a good fit, between 0.05 and 0.08 an acceptable fit, and greater than 0.8 a mediocre fit, whereas values > 0.10 are not acceptable.

Changes in goodness-of-fit statistics were also examined to detect differences in the different models. A significant difference in *χ*^2^ values between nested models means that all equality constraints do not hold across the groups.

Graphical representation of the GAS items measured on an ordinal scale shows that normality assumption is not tenable. As a consequence, asymptotically distribution-free estimation instead of maximum likelihood estimation is a good strategy to accommodate non-normally distributed data in SEM analyses.

Lastly, concurrent validity was investigated by correlating the total GAS score with the scores of the MDI [53]; the BSSS [57]; and the Neuroticism-Anxiety, Sociability, and Aggression-Hostility subscales of the ZKPQ-50-cc [58]. We also examined the strength of the association of the scale with other measures related to alcohol and cannabis use. According to Cohen’s rule of thumb, any correlation greater than 0.5 is large, from 0.5-0.3 is moderate, from 03–0.1 is small, and less than 0.1 is trivial [68].

#### Missing values

GAS missing values were handled with the hot deck imputation method, in which each missing value is replaced with an observed response from a similar unit with respect to the characteristics observed by both cases [69]. In our study, the BSSS was chosen as the “deck variable,” as it includes little to no missing data [70]. We used a hot deck imputation macro for SPSS users by T. van der Weegen, which can be downloaded from the following website: http://www.spsstools.net/SampleSyntax.htm.

#### Sample size considerations

Sample size plays an important role in providing unbiased parameter estimates and accurate model fit information. Following Bentler and Chou [71], who recommended at least a 5:1 ratio of subjects to variables for normal and elliptical distributions, there seems to be a general consensus among researchers for the adoption of this ratio. However, for categorical or non-normally distributed variables, as is the case here, larger samples are required than for continuous or normally distributed variables. A ratio of at least 10 subjects per variable for this type of distribution is recommended [72]. The sample in the present study fulfills this requirement.

## Results

Of the original 5,990 observations initially recorded, GAS data were missing for 42 participants (0.7 %). The use of hot deck imputation successfully imputed data for 35 of them, still leaving 7 cases incomplete. A final sample size of 5,983 respondents (3,318, French-speaking and 2,665 German-speaking) was then analyzed. Participants’ mean age was 20.0 years (SD = 1.2). Of this final sample, 10.6 % of the French and 8.1 % of the German respondents were classified as polythetic users, whereas 2.3 % of respondents in each group were classified as monothetic users. The characteristics of each linguistic region are reported in Table 2.

### French-speaking community

The internal consistency of the GAS was good, as reflected by a Cronbach’s coefficient of 0.86. EFA by Velicer’s MAP test suggested a one-factor solution. This finding was successfully confirmed by parallel analysis. This one-factor model was then evaluated in CFA with AMOS. Guided by modification indices and unusual standardized residuals that suggested the correlation of six error variances, we established a well-fitted model that exhibited good fit relative to the independence model (*χ*^2^/df = 2.6, CFI = 0.99, RMSEA = 0.02).

### German-speaking community

The internal consistency of the scale was satisfactory (Cronbach α = 0.85). A one-factor solution was also found in EFA by Velicer’s MAP and was confirmed by parallel analysis. The same path model used to evaluate the French-speaking group was applied to the German-speaking group. This model performed more poorly but still gave acceptable goodness-of-fit values (*χ*^2^/df = 5.9, CFI = 0.94, RMSEA = 0.04).

### Multigroup analysis

#### Testing for configural equivalence

Having determined a well-fitting model for each group separately, we tested configural equivalence in which the same parameters were estimated again in a multigroup model. In other words, parameters were estimated for both groups at the same time. Results related to this multigroup model revealed a *χ*^2^ value of 91.53 with 17 degrees of freedom. The CFI and RMSEA values were 0.97 and 0.02, respectively, providing an acceptable fit. These values are the baseline values against which all subsequent tests for invariance were compared.

#### Testing for factorial measurement equivalence

A model with all loadings (factor loadings by group are displayed in Table 3) constrained to be equal across groups was fitted. Goodness-of-fit statistics related to this constrained two-group model are presented in Table 4 (second entry). In testing for the invariance of this constrained model, we compared its *χ*^2^ value of 114.59 with 23 degrees of freedom with that for the unconstrained model (*χ*^2^_(17)_ = 91.53). This comparison yielded a *χ*^2^ difference (Δχ^2^) of 23.06 with 6 degrees of freedom, which is statistically significant (*p =* 0.001). Hence, the equality constraints for all factor loadings were rejected. Given the rejection of full factorial invariance, we proceeded to check which factors loading were different. As factor-loading parameters were found to be invariant across groups, their specified equality constraints were maintained, cumulatively, throughout the remainder of the invariance-testing process [73]. First, constraining factor loadings of the Tolerance item to be equal across groups yielded non-significant results, suggesting they are equal. For identification purposes, loading for the Salience item was already constrained to take the value of 1 in both groups. Next, holding this equality constraint and adding the equality constraint for Mood Modification still resulted in non-significant *χ*^2^ values. This continued until we reached Withdrawal, where significant *χ*^2^ results suggested non-equality between the two groups. Tests were repeated for Conflict and Problems, which were again non-significant. The detailed procedure is shown in Table 4. All observed measures except for Withdrawal were found to be operating equivalently for both linguistic regions.

**Table 3:** Factor loadings and goodness-of-fit measures

		Factor loading	
		French-speaking	German-speaking
Item 1	Salience	0.65	0.62
Item 2	Tolerance	0.65	0.65
Item 3	Mood modification	0.73	0.72
Item 4	Relapse	0.73	0.71
Item 5	Withdrawal	0.65	0.71
Item 6	Conflict	0.64	0.62
Item 7	Problems	0.65	0.61
Cronbach α		0.86	0.85
*χ* ^2^/df		2.6	5.9
RMSEA		0.02	0.04
CFI		0.99	0.94

**Table 4:** Summary of goodness-of-fit statistics for tests of invariance across linguistic groups

Model Description	*χ* ^2^	df	Δχ^2^	Δdf	Statistical significance
1. Unconstrained model	91.53	17	-	-	-
2. All factor loadings constrained equally across groups	114.59	23	23.06	6	*p =* 0.001
Model 2.1	91.92	18	0.39	1	*p =* 0.5
Model 2.2	92.10	19	0.56	2	*p =* 0.8
Model 2.3	95.85	20	4.32	3	*p =* 0.2
Model 2.4	105.52	21	13.99	4	*p =* 0.007
Model 2.5	95.90	21	4.37	4	*p =* 0.4
Model 2.6	99.37	22	7.84	5	*p =* 0.2

Model 2.1: Tolerance loadings equal between French and German-speaking groups

Model 2.2: Tolerance and Mood Modification loadings equal

Model 2.3: Tolerance, Mood Modification and Relapse loadings equal

Model 2.4: Tolerance, Mood Modification, Relapse and Withdrawal loadings equal

Model 2.5: Tolerance, Mood Modification, Relapse and Conflict loadings equal

Model 2.6: Tolerance, Mood Modification, Relapse, Conflict and Problems loadings equal

#### Correlation analysis in the French-speaking community

Correlation analysis was used to explore concurrent validity between GAS and other similar constructs. As shown in Table 5, the association of GAS with the MDI total score and with the ZKPQ-50-cc Anxiety subscale was small (ρ = 0.27 and ρ = 0.24, respectively) and the association of GAS with the ZKPQ-50-cc Sociability subscale was small and negative (ρ = −0.20). The correlations with the other assessment measures were considered trivial.

**Table 5:** Correlation between GAS and other constructs in the French-speaking community (among Fra)

	1	2	3	4	5	6	7	8	9	10	11	12	13	14
1. GAS	1.00													
2. MDI	0.27	1.00												
3. BSSS (full scale)	0.01	0.09	1.00											
4. Experience seeking (subscale)	0.04	0.10	0.79	1.00										
5. Boredom (subscale)	−0.03	0.11	0.78	0.54	1.00									
6. Adventure (subscale)	−0.02	0.001	0.77	0.43	0.42	1.00								
7. Disinhibition (subscale)	0.05	0.08	0.79	0.48	0.51	0.50	1.00							
8. Aggressivity ZKPQ-50-cc	0.09	0.14	0.21	0.07	0.18	0.16	0.25	1.00						
9. Sociability ZKPQ-50-cc	−0.20	−0.18	0.13	−0.01	0.10	0.10	0.22	0.03	1.00					
10. Anxiety ZKPQ-50-cc	0.24	0.46	0.02	0.05	0.08	−0.09	0.01	0.15	−0.25	1.00				
11. Binge: 6 alcoholic beverages at one sitting	0.01	−0.08	−0.33	−0.18	−0.22	−0.17	−0.46	.0.16	−0.21	−0.01	1.00			
12. Frequency of drinking in days per week	−0.05	0.05	0.24	0.14	0.19	0.11	0.31	0.12	0.13	0.02	−0.61	1.00		
13. Age cannabis use onset	0.07	−0.06	−0.20	−0.13	−0.15	−0.14	−0.18	−0.08	0.01	−0.02	0.11	−0.9	1.00	
14. Age getting “high” on cannabis for the first time	0.06	−0.06	−0.18	−0.11	−0.15	−0.13	−0.17	−0.09	0.01	−0.02	0.08	−0.9	0.93	1.00

#### Correlation analysis in the German-speaking community

As shown in Table 6, the association of GAS with MDI and with the ZKPQ-50-cc Anxiety subscale was small (ρ = 0.24 and ρ = 0.23). This association was smaller with the ZKPQ-50-cc Aggressivity subscale (ρ = 0.15) and with the Sociability subscale (ρ = − 0.10).

**Table 6:** Correlation between GAS and other constructs in the German-speaking community

	1	2	3	4	5	6	7	8	9	10	11	12	13	14
1. GAS	1.00													
2. MDI	0.24	1.00												
3. BSSS (full scale)	0.01	0.02	1.00											
4. Experience seeking (subscale)	−0.02	0.03	0.78	1.00										
5. Boredom (subscale)	−0.03	0.04	0.79	0.55	1.00									
6. Adventure (subscale)	0.02	−0.04	0.79	0.44	0.45	1.00								
7. Disinhibition (subscale)	0.06	0.02	0.79	0.44	0.51	0.54	1.00							
8. Aggressivity ZKPQ-50-cc	0.15	0.16	0.19	0.03	0.16	0.13	0.27	1.00						
9. Sociability ZKPQ-50-cc	−0.10	−0.20	0.21	0.05	0.18	0.14	0.30	0.09	1.00					
10. Anxiety ZKPQ-50-cc	0.23	0.45	−0.04	−0.04	0.02	−0.10	0.01	0.26	−0.25	1.00				
11. Binge: 6 alcoholic beverages at one sitting	−0.05	−0.04	−0.27	−0.10	−0.17	−0.19	−0.40	−0.17	−0.23	−0.01	1.00			
12. Frequency of drinking in days per week	0.02	0.10	0.18	0.06	0.14	0.10	0.26	0-14	0.13	0.04	−0.57	1.00		
13. Age cannabis use onset	−0.03	−0.08	−0.12	−0.02	−0.06	−0.11	−0.19	−0.17	−0.02	0	0.19	−0.21	1.00	
14. Age getting “high” on cannabis for the first time	−0.03	−0.08	−0.10	−0.02	−0.04	−0.10	−0.16	−0.16	0.004	0.01	0.16	−0.18	0.90	1.00

## Discussion

The present study is the first to assess, to our knowledge, the psychometric characteristics of the 7-item GAS among representative samples of French- and German-speaking adult men.

The main finding is that the one-factor model of the 7-item GAS has good psychometric properties and fits the data well in both samples. The results are in accordance with a number of previous findings [44, 50] and allow their extension to adults. [74, 75].

Furthermore, all observed measures except for Withdrawal were found to be operating equivalently for both linguistic regions. This adds to the cross-linguistic validity of the scale. The weakness related to the Withdrawal-related item may be due to lack of precision of this concept when applied to game use [36]. It may also indicate cross-group differences in the underlying construct. This hypothesis does not hold, however, because these differences are not reflected in the magnitude of the factor loadings, whose values are similar (0.65 vs. 0.71). Discrepancies between the French and the German translation of this related item may explain this difference. However, after discussing this again with bilingual individuals, we cannot find major discrepancies in the meaning of the words used. Although this is the largest difference in factor loadings, it remains marginal compared with the others (0.06 in absolute value). Hence, the only plausible explanation is that the statistical significance of the *χ*^2^ statistics observed is in all likelihood induced by the large sample size of almost 6,000 individuals.

In concordance with numerous studies on game and Internet use [19, 21, 76], an association was found between depressive symptoms and GAS scores. In addition, a small association was found between GAS scores and both the Neuroticism-Anxiety dimension and the Aggression-Hostility subscale of the ZKPQ-50-cc. These associations are in line with findings related to substance use-related addictions [77, 78] and are in concordance with other studies related to Internet or game addiction [59, 79]. Moreover, as in other studies [79], a negative association was found with the Sociability subscale. This seems to be consistent with the findings of other studies that showed an association between loneliness and low social competence with game addiction [25, 80].

The present study did not show an association between GAS scores and sensation seeking. This finding contradicts that of other studies [81]. Some researchers have shown that sensation seeking is related to extraversion [58]. However, game and Internet addictions seem to be more linked to introversion than to extraversion [82], and so it is plausible that sensation seeking was not associated here with the GAS scores. Similarly, in contradiction to the findings of a number of previous studies [19, 26, 32, 83], the present study failed to show an association with alcohol or cannabis use. These associations were possibly mediated by the specific preferred online activity and may differ from one activity to another [84].

With an overall 2.3 % of participants classified as monothetic users and an additional 9.5 % classified as polythetical users (excessive users), the prevalence rates in this study are comparable with those found in the initial GAS study [44] and in a number of other Swiss and European studies [85–89]. Slightly lower [90, 91] or higher prevalence figures [12, 92] were, however, reported in other studies. The differences are probably a consequence of differences in assessment tools, population studied, use of polythetic classification, and proposed cutoffs [12].

The study has a number of strengths, such as the recruitment of a representative sample of young men and a high response rate. This is a possible advantage in consideration of the self-selection bias described in online recruitment-based studies [93]. Another important strength is the inclusion of two different and large linguistic samples. Among the weaknesses of the study are a lack of women in the present samples and a lack of concomitant evaluation of the specific games activities of the participants. Further studies of the GAS may be needed to assess different games and other Internet-related behaviors.

## Conclusion

The 7-item GAS seems to be an interesting assessment tool. This scale, previously used for adolescent samples, appears to be adequate for adult samples and has good psychometric properties in its French and German versions.

### Ethics approval and consent to participate

The study at hand, issued from the C-SURF research protocol number 15/07, was approved by the Lausanne University Medical School's Ethics Committee for Clinical Research. All participants gave their written informed consent to participate in the study.

### Consent for publication

Not applicable.

### Availability of data and materials

Availble upon requesto to the last author Gerhard Gmel: Gerhard.Gmel@chuv.ch.

## Additional file

Additional file 1: Translation of the Game Addiction Scale (DOCX 72 kb)

## Abbreviations

BSSS: brief sensation seeking scale
CFA: confirmatory factor analysis
CFI: comparative fit index
C-SURF: cohort study on substance use risk factors
DSM-IV: diagnostic statistical manual of mental disorders, fourth edition
EFAs: exploratory factor analyses
GAS: game addiction scale
ICD-10: international classification of mental and behavioral disorders
MAP: velicer’s minimum average partial test
MDI: major depression inventory
RMSEA: root mean square error of approximation
SEM: structural equation modeling
ZKPQ-50-cc: Zuckerman-Kuhlman personality questionnaire
