# Supplementary material for: Psychometric properties of the 7-item game addiction scale among french and German speaking adults
Source: BMC Psychiatry. 2016 May 10;16:132. doi: 10.1186/s12888-016-0836-3 (PMC4862221; doi:10.1186/s12888-016-0836-3)
Supplement: Additional file 1: — Translation of the Game Addiction Scale (DOCX 72 kb) [file 12888_2016_836_MOESM1_ESM.docx]

**French Translation of the Game Addiction Scale (GAS) [1]**

*Les prochaines questions portent sur les jeux online (Internet), sur le temps que vous passez sur Internet pour jouer et sur les jeux électroniques (Nintendo, Playstation, X-Box, Wii, PC, etc.)*

**Au cours des six derniers mois, à quelle fréquence…**

Cochez une case par ligne.

|  | Jamais | Rarement | Parfois | Souvent | Très souvent |
| --- | --- | --- | --- | --- | --- |
| …avez-vous pensé à jouer? |  |  |  |  |  |
| …avez-vous joué plus longtemps que prévu? |  |  |  |  |  |
| …avez-vous joué à des jeux pour oublier votre vie quotidienne? |  |  |  |  |  |
| ... est-ce que d’autres personnes de votre entourage ont essayé, sans succès, de vous faire réduire le temps que vous passez à jouer ? |  |  |  |  |  |
| …vous êtes-vous senti mal quand vous ne pouviez pas jouer? |  |  |  |  |  |
| ... avez-vous eu des conflits avec des membres de votre entourage (famille, amis) en raison du temps que vous passez à jouer ? |  |  |  |  |  |
| ...avez-vous négligé d’autres activités pour jouer? |  |  |  |  |  |

**German Translation of the Game Addiction Scale (GAS) [1]**

In den nächsten Fragen geht um die Zeit, die Sie mit „Gamen“ verbracht haben. Dies beinhaltet das Spielen von Cybergames im Internet und auch auf Spielkonsolen (z.B. Nintendo, Playstation, X-Box, Wii).

**Wie oft in den letzten 6 Monaten...**

Kreuzen Sie in jeder Zeile ein Kästchen an

|  | Nie | Selten | Manch-mal | Oft | Sehr of |
| --- | --- | --- | --- | --- | --- |
| … ...haben Sie den ganzen Tag ans 'gamen' gedacht? |  |  |  |  |  |
| …...haben Sie länger 'gegamet' als ursprünglich beabsichtigt? |  |  |  |  |  |
| ... haben Sie 'gegamet', um Ihren Alltag zu vergessen? |  |  |  |  |  |
| ... haben andere vergeblich versucht, Sie dazu zu bringen, weniger zu 'gamen' zu verbringen? |  |  |  |  |  |
| ... haben Sie sich schlecht gefühlt, wenn Sie nicht 'gamen' gehen konnten? |  |  |  |  |  |
| ... haben Sie Streit gehabt mit anderen (z.B. Familie, Freunde) wegen der Zeit, die Sie mit 'gamen' verbrachten? |  |  |  |  |  |
| ...haben Sie wichtige Aktivitäten (z.B. Schule, Arbeit, Sport) vernachlässigt, um 'gamen' zu können zu sein? |  |  |  |  |  |

1. Lemmens JS, Valkenburg PM, Peter J: **Development and Validation of a Game Addiction Scale for Adolescents**. *Media Psychology* 2009, **12**(1):77-95.
